# Validation of antibodies: Lessons learned from the Common Fund Protein Capture Reagents Program

**DOI:** 10.1126/sciadv.abl7148

**Published:** 2021-11-10

**Authors:** Ananda L. Roy, Elizabeth L. Wilder, James M. Anderson

**Affiliations:** 1 Office of Strategic Coordination, National Institutes of Health, Bethesda, MD 20892, USA.; 2 Division of Program Coordination, Planning and Strategic Initiatives, Office of the Director, National Institutes of Health, Bethesda, MD 20892, USA.

## Abstract

Large-scale generation of protein capture reagents remains a technical challenge, but their generation is just the beginning. Validation is a critical, iterative process that yields different results for different uses. Independent, community-based validation offers the possibility of transparent data sharing, with use case–specific results made broadly available. This type of resource, which can grow as new validation data are obtained for an expanding group of reagents, provides a community resource that should accompany future reagent-generating efforts. To address a pressing need for antibodies or other reagents that recognize human proteins, the National Institutes of Health Common Fund launched the Protein Capture Reagents Program in 2010 as a pilot to target human transcription factors. Here, we describe lessons learned from this program concerning generation and validation of research reagents, which we believe are generally applicable for future research endeavors working in a similar space.

## Teaser

Large-scale validation is extremely challenging as validation remains “in the eye of the beholder.”

## BACKGROUND

Antibodies play a pivotal role in biomedical research as well as in diagnostics and therapeutic applications (*1*). In biomedical research, protein-based biochemical assays [e.g., Western blot and immunoprecipitation (IP)], cell-based assays (e.g., enzyme-linked immunosorbent assay and flow cytometry), and increasingly imaging assays (e.g., immunohistochemistry) and proteomic assays (e.g., CODEX and CyTOF) use antibodies (*2*–*4*). With expected advancements in proteomic analysis including single-molecule protein sequencing and fingerprinting techniques, these methods will certainly lead to a better coverage and identification of functional proteoforms in tissues under distinct conditions (*5*). In diagnostics, detection of various markers and therapeutic applications (a recent example being the monoclonal antibody cocktail used in COVID-19) of various forms of antibodies are increasingly reminding us of the value of these reagents. Given that monoclonal antibodies are large (150 kDa), their stability and cell permeability could pose a problem. A series of next-generation derivatives like recombinant antibodies, engineered non-immunoglobulin protein scaffolds, single-chain Fv, or variants like diabodies and minibodies (molecular weight ranging from 25 to 100 kDa) and lastly camelids or nanobodies (~12 to 16 kDa) have come into existence and find increasing use (*6*–*8*). Although each of these reagents is useful for a set of specific applications, polyclonal and monoclonal antibodies remain the workhorse in the field of protein detection, particularly in laboratory settings (*9*). Despite the extensive use of antibodies, validated antibodies for community use remain a vexing problem. This is in large part because assessing whether an antibody is right for the intended purpose involves determining its target specificity accurately and at the same time ascertaining whether it is suitable for the particular purpose. In addition, standardized procedures and protocols for specific use cases are critical to ensure quality of experimental approaches (*10*) yet can differ among laboratories. Thus, a one-size-fits-all reagent for every application and for every end use is simply unachievable.

**Fig. 1.**
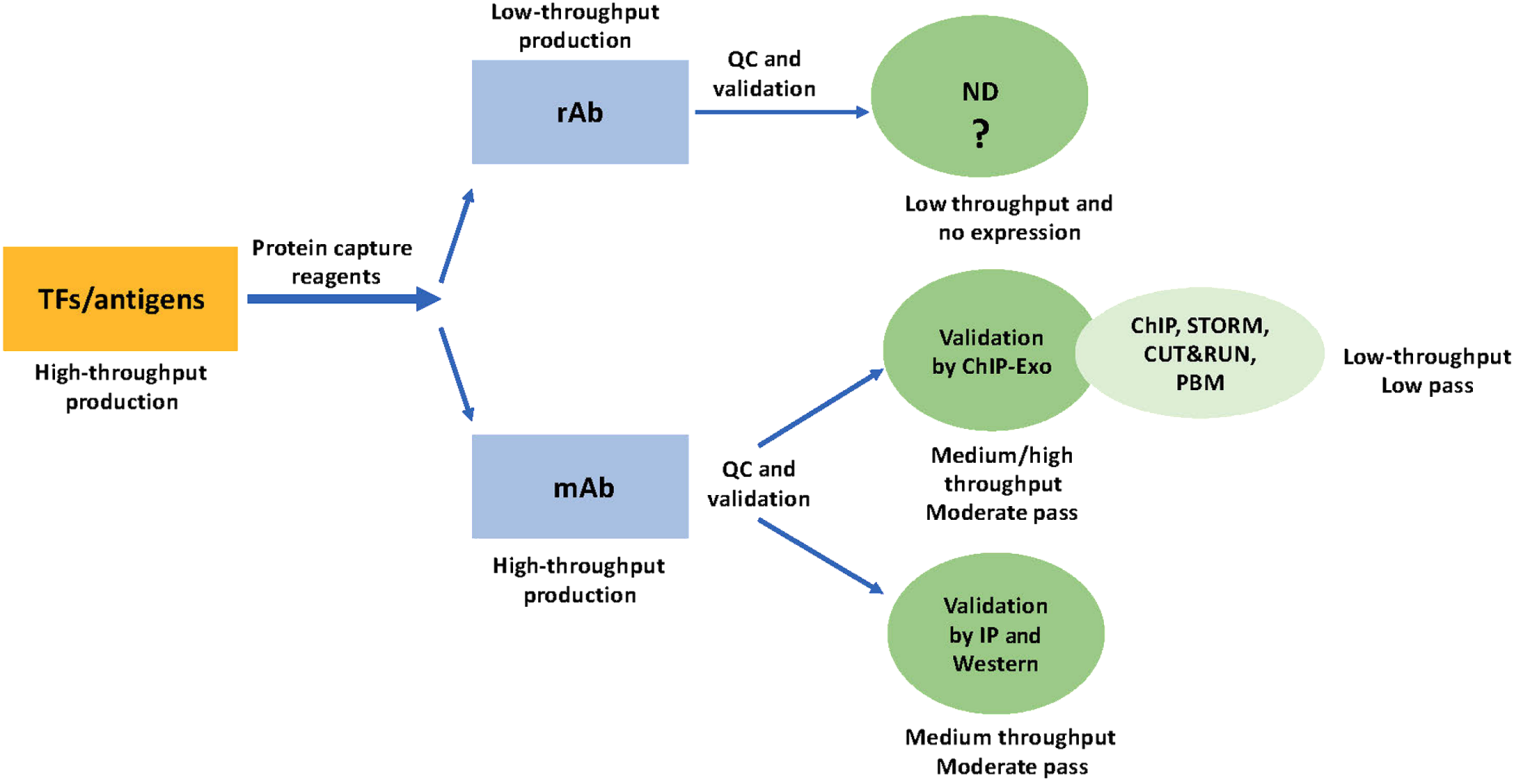
Schematics of the PCRP program. Generation of human transcription factor (TFs) antigens (as recombinant GST-tagged protein fragments) in bacteria was a high-throughput endeavor. Generation of capture reagents was divided into two groups: as either recombinant antibody, rAb (low throughput) or monoclonal antibody, mAb (high throughput). rAb-ND—not determined as no antibody expression was detected. So, no assay could be done. The reasons for this remain unknown. The mAbs were validated in a medium-throughput fashion by both Western blot and IP with a moderate pass rate. The recent effort by Lai *et al.* describes a medium-/high-throughput approach to validate around 900 mAbs by ChIP-Exo with a moderate pass rate, and a subset of these were used in ChIP, STORM, CUT&RUN, and PBM assays with a low pass rate. With so many use cases, each community will have to validate for that particular technology and even specific platforms for their own specific purpose.

Although antibodies have been essential reagents for biomedical research and clinical diagnostics for decades (*11*–*15*), a major challenge the biomedical research community perpetually faces is the lack of highly specific, renewable, and validated protein capture reagents. It is estimated that more than 300 companies sell more than 2 million antibodies globally for research with the market worth in excess of $1.5 billion, according to global consultancy Frost & Sullivan. Yet, irreproducibility of results in a substantial number of biomedical-research publications, particularly those using antibodies, is a recurring problem (*11*–*15*). The irreproducibility could arise because of several factors, including the source and nature of the reagents, validation methods, investigator-dependent protocol differences, and general lack of consensus industry standards. The provenance of a reagent might be undocumented if sourced from another company or an investigator. The operational definition of validation provided by the Federal Government Agency Food and Drug Administration (FDA) is “the process of demonstrating, through the use of specific laboratory investigations, that the performance characteristics of an analytical method are suitable for its intended analytical use” (www.fda.gov/downloads/Drugs/GuidanceComplianceRegulatoryInformation/Guidances/UCM070107.pdf). In addition, the International Working Group for Antibody Validation suggests five different “pillars” to be applied for proper validation (*10*). For antibodies, validation criteria must minimally include target specificity, assay selectivity, and lastly reproducibility (*10*). Last, if the reagents are not renewable, once the primary source is depleted or production is stopped for any number of reasons, these reagents become permanently unavailable (*11*–*15*). For this reason, although polyclonal antibodies have been extremely useful for biomedical research (*16*), once they are used up, a new “bleed” and/or a different lot might not have the same properties as the original reagent.

To address these concerns, a workshop was conducted in 2010 by the NIH to gather information on the state of the art in technology for high-throughput production of renewable and high-quality affinity reagents for the human proteome. Although monoclonal antibodies are in theory renewable and presented a mature methodology, with clear downstream applications already developed, they were deemed to have drawbacks that posed a practical barrier to creating a more comprehensive set of protein capture reagents. Thus, in addition to monoclonal antibodies, a range of alternative technologies such as recombinant antibodies, engineered proteins, and aptamers were considered. Further considerations were given to emerging approaches for the generation of protein affinity capture reagents that might be scalable to the entire proteome and made available for wide use by the research community.

The limitations and advantages of each approach and consideration of which of these could productively go forward at a “production level” were discussed. Given that the rate of discovery of new proteins by various methods far exceeds the commercial antibody supply, there was a clear need to generate these protein capture reagents in a high-throughput fashion. A further consideration would be the antigens—microgram quantities of purified antigen source (full-length protein, fragments, or synthetic peptides) are often needed to generate and validate antibodies by binding assays (*17*). In addition, the antigenic source should be pure, ideally retain its native conformation, and be soluble (*17*). Hence, a central question that the workshop participants grappled with was the idea of a “comprehensive” set of capture reagents at scale. Furthermore, would a comprehensive set of reagents need to recognize posttranslationally modified proteins and numerous splice-forms and would multiple epitopes per protein need to be covered? The potential scope was almost limitless.

Given the growing need for affinity reagents, the Common Fund undertook the challenge to address this “field-limiting” area of biomedical research by launching the Protein Capture Reagents Program (PCRP) in 2010 to support technology development for new classes of reagents and further to streamline antibody production so that these capture reagents could be generated at scale. A major goal of the National Institutes of Health (NIH) Common Fund program is to address emerging scientific opportunities and pressing challenges in biomedical research that no single NIH Institute or Center is poised to address on its own. Common Fund–supported programs are intended to change paradigms, develop innovative tools and technologies to change the trajectory of a field, and/or provide foundations for research that can be used by the broad biomedical research community. These goal-driven programs are developed to catalyze a particular biomedical field of research within a span of 5 to 10 years.

One aim of the PCRP was to support the development of novel reagent classes that could potentially be scalable to the entire proteome. A second aim was to attempt to adapt antibody production so that high-throughput methods might be feasible. For the second aim, human transcription factors were selected as an initial group of targets. If either of these aims was successful in the first 5 years of the program, the goal for a second 5 years would be to scale the new approaches to the proteome (https://commonfund.nih.gov/proteincapture). The PCRP’s antibody production effort included two parallel approaches: monoclonal antibody production and generation of recombinant antibodies (Fig. 1). Both antibody-producing components were supported by a common antigen-producing component (*18*, *19*). The output of this program has been described, along with initial validation of the reagents (*20*, *21*). However, the lack of uptake of these reagents by the broader community led us to conclude that additional validation was required. Recognizing that investigators with specific use requirements would be in the best position to validate the reagents for their uses, we issued a supplemental funding opportunity to NIH awardees who would be interested and able to validate PCRP antibodies for different uses. The results of these efforts are reported in a recently published paper by Lai *et al.* (*22*).

## IMPORTANCE OF INDEPENDENT VALIDATION FOR SPECIFIC USES

The PCRP effort to generate monoclonal antibodies targeting all human transcription factors yielded a collection of 1406 mouse monoclonal antibodies to 737 human transcription factors, which have been made available to the research community at a fraction of the cost of commercially available antibodies (*20*, *21*). In addition, several hundred recombinant antibodies were generated (Fig. 1). Although the NIH promoted use of the reagents through various communication channels, the portal through which information on the antibodies is made available showed minimal traffic. While this may indicate that outreach efforts were unsuccessful, feedback from investigators representing likely users of these reagents indicated that the validation data for the reagents did not address their specific uses. For instance, each antibody (either monoclonal or recombinant) was subjected to a high-throughput initial screen of either affinity or specificity and only sent for further validation if it passed this screen. Each reagent that passed this primary screen was then subjected to secondary validation, which included IP, spiked-IP, and/or Western blotting [(*20*); https://proteincapture.org/about/ran/]. However, focus group discussions with end users and responses submitted through a request for information yielded a consensus view that validation by chromatin IP (ChIP) is a common requirement for the transcription factor research community.

Large-scale antibody validation efforts are rare, given that no single assay can measure the utility of antibodies across the spectrum. A large-scale validation of antibodies only for Western blot applications has been described, which validated more than 6000 antibodies by at least one of the strategies (*23*–*25*). A notable effort in this realm is the validation and targeted proteomics approach undertaken by the National Cancer Institute’s Clinical Proteomic Tumor Analysis Consortium, which is a comprehensive and well-coordinated IP–mass spectrometry (MS)–based effort to elevate rigor and reproducibility (*26*). Another systematic initiative focusing on mapping the entire human proteome via MS is the Human Proteome Project launched by the Human Proteome Organization (*27*, *28*). Likewise, using a set of orthogonal approaches, a recent study characterized the spatial distribution of proteins in single cells of complex tissue samples via antibodies (*23*). They validated 5981 antibodies that recognized expression of 3775 human proteins across all major human tissues and uncovered 56 proteins corresponding to the group of “missing” proteins and 171 proteins of unknown function or so-called “dark” proteins (*23*). Last, the Human Protein Atlas has a large number of datasets characterizing protein expression by immunohistochemistry (*23*). This is perhaps the largest coordinated effort with the goal for antibody-based targeting of the entire human proteome, with coverage of more than 15,000 proteins corresponding to nearly 80% of the protein-coding genome (*23*). However, a functional approach to validate a large set of antibodies against transcription factors in a high-throughput fashion by using ChIP has not been undertaken before.

To fill this gap, Lai *et al.* (*22*) have primarily used a derivative of ChIP, ChIP-Exo, for their individual uses to validate a large collection of monoclonal antibodies and have collaborated to report results. A more limited set of antibodies were also validated by additional methods, including ChIP, protein binding microarray, and high-resolution imaging (*22*) (Fig. 1). An important conclusion drawn is that validation for one use does not equate to validation for another use. While the current report will be valuable to researchers using the assays described, users studying transcription factors through other assays may find other antibodies within the PCRP collection that work for them. Independent validation of reagents by different research groups using different assays yields a richer validation dataset.

Another critical validation consideration is the threshold for determining that the reagent “passes” the validation step. As noted by Lai *et al.* (*22*), relaxed criteria for validation would render more antibodies “passing” compared to a more stringent criterion. However, regardless of the threshold, an antibody that “fails” in a particular assay might prove to be useful in another one. The manner in which an antibody fails also requires consideration. None of the recombinant antibodies that were explored in the study passed the validation assays because none could be expressed. These antibodies must be purified from bacterial expression constructs that may present a challenge for some investigators, but collectively, Lai *et al.* have a long-standing experience in protein purification. The failure of these reagents in the various assays may therefore represent a problem with the reagents’ ability to function in the assays performed in this study. Another type of “fail” occurs in the reported assays when a reagent captures a protein that does not behave as expected. Transcription factors can recognize well-established cognate sequences directly or they may recognize yet unknown sequences via indirect interactions. Thus, the binding of an antibody to a transcription factor that recognizes an unexpected sequence could indicate nonspecificity of the antibody or it could imply novel biology—this is especially true for factors with little known biology to begin with (so-called dark proteins). Since the provision of reagents to elucidate the functions of understudied proteins was a goal for the PCRP, the validation experiments that show this type of result are particularly intriguing. It is also worth considering the fact that human protein antigen derivatives were used to immunize mice, which might not elicit a strong immunogenic reaction given the high degree of conservation of transcription factors between these species. Whether this led to relatively low rate of success in validation efforts remains unknown. Last, it is also worth considering whether such large-scale protein-based approaches will ever scale given the highly variable and unpredictable chemistry of peptides and proteins (solubility, oxidation states, etc.) compared with the relative simplicity of nucleic acids. This is another reason to continue investigating more predictable capture reagents such as aptamers.

A final validation consideration is the method through which validation analyses are reported (*1*, *15*). The specific conditions for any validation assay will have substantial effects on the outcome, so sharing the detailed experimental methods for reagent validation is essential. The Lai *et al.* validation study describes a collaborative effort to make validation data public so that it can be referenced and reproduced, or if it is not reproducible, so that it can be adjusted (*22*), as the publicly available portal is able to accept new validation data from the community as more users use these antibodies in different assays in their own laboratory setting. Hence, this validation site represents a significant deliverable from the PCRP program as a source of easily findable, accessible, and extendable validation data (Fig. 1).

## PERSPECTIVE ON PCRP AND PROTEIN CAPTURE REAGENTS TODAY

PCRP was well suited for the Common Fund. It addressed a substantial barrier for biomedical researchers working in basic and clinical research, across the NIH mission. It had extremely high-risk objectives. The fact that the program was not successful in developing reagents for the entire proteome reflects the difficulty of the goals. The validation efforts reported by Lai *et al.* are an important coda to the program, and we hope it will spur greater use of the reagents the program delivered. Although the PCRP raises a cautionary tale for high-throughput approaches to functionally validate capture reagents, it is difficult to predict whether this sort of large-scale endeavor will be successful for any specific class of proteins other than transcription factors, underscoring the inherent difficulties in such risky ventures.

The use case–specific requirements for validation present a challenge for any large-scale reagent generation program that might be considered in the future. The generation of capture reagents may ultimately be scalable, but validation is likely to remain a low-throughput endeavor for many, if not most, applications. Validation is, to some extent, “in the eye of the beholder” and is therefore best suited for approaches such as described in Lai *et al.* (*22*)—carried out by individual laboratories for individual purposes depending on the function of the target antigen(s) to be tested. Although this type of validation is essential for rigor and reproducibility of experiments, it is not amenable to a single protocol. Thus, we conclude that with so many use cases, each community will have to validate for that particular technology and even specific platforms for their own specific purpose. To enhance rigor and reproductivity of NIH-supported research, applicants for funding are required to explain how they intend to authenticate key biological and/or chemical resources including antibodies. In this context, it is perhaps best for individual laboratories to make the validation data available to the broad scientific community for real-time input either through publicly accessible data portals or through open access publications. The results provided by Lai *et al.* strongly highlight the need for investigators to devise the most appropriate validation assays for their specific purposes, make the pass/fail criteria for these assays transparent, and make the data public.
